# Meta-analysis of heterogeneous Down Syndrome data reveals consistent genome-wide dosage effects related to neurological processes

**DOI:** 10.1186/1471-2164-12-229

**Published:** 2011-05-11

**Authors:** Mireia Vilardell, Axel Rasche, Anja Thormann, Elisabeth Maschke-Dutz, Luis A Pérez-Jurado, Hans Lehrach, Ralf Herwig

**Affiliations:** 1 Department of Vertebrate Genomics, Max-Planck-Institute for Molecular Genetics, Ihnestr. 63-73, D-14195 Berlin, Germany; 2 Unitat de Genètica, Universitat Pompeu Fabra, y CIBER de Enfermedades Raras (CIBERER), Parc de Recerca Biomèdica de Barcelona, C/Dr Aiguader, 88, 08003, Barcelona, Spain; 3 Programa de Medicina Molecular I Genetica, Hospital Vall d'Hebron, 08035 Barcelona, Spain

## Abstract

**Background:**

Down syndrome (DS; trisomy 21) is the most common genetic cause of mental retardation in the human population and key molecular networks dysregulated in DS are still unknown. Many different experimental techniques have been applied to analyse the effects of dosage imbalance at the molecular and phenotypical level, however, currently no integrative approach exists that attempts to extract the common information.

**Results:**

We have performed a statistical meta-analysis from 45 heterogeneous publicly available DS data sets in order to identify consistent dosage effects from these studies. We identified 324 genes with significant genome-wide dosage effects, including well investigated genes like *SOD1*, *APP*, *RUNX1 *and *DYRK1A *as well as a large proportion of novel genes (N = 62). Furthermore, we characterized these genes using gene ontology, molecular interactions and promoter sequence analysis. In order to judge relevance of the 324 genes for more general cerebral pathologies we used independent publicly available microarry data from brain studies not related with DS and identified a subset of 79 genes with potential impact for neurocognitive processes. All results have been made available through a web server under http://ds-geneminer.molgen.mpg.de/.

**Conclusions:**

Our study represents a comprehensive integrative analysis of heterogeneous data including genome-wide transcript levels in the domain of trisomy 21. The detected dosage effects build a resource for further studies of DS pathology and the development of new therapies.

## Background

Down syndrome (DS) is the most frequent genomic aneuploidy with an incidence of approximately 1 in 700 live-newborn [1] resulting from the presence of an extra copy of human chromosome 21 (HSA21). DS is characterized by a complex phenotype with features that are not fully penetrant. The most frequent manifestations, which are virtually always present, include mental retardation, morphological abnormalities of the head and limbs, short stature, hypotonia and hyperlaxity of ligaments. Other features occur with less frequency such as organ malformations, particularly of the heart (50% of DS newborns), several types of gastrointestinal tract obstructions or dysfunctions (4-5% of DS newborns), increased risk of leukaemia (20 × higher compared to the normal population), and early occurrence of an Alzheimer-like neuropathology [2,3]. DS has been investigated with multiple functional genomics studies aiming to understand the molecular basis underlying the various aspects of the disease [4-7].

The most commonly accepted pathogenetic hypothesis is that the dosage imbalance of genes on HSA21 is responsible for the molecular dysfunctions in DS, meaning that genes on the triplicated chromosome are overexpressed due to an extra chromosome 21, as demonstrated for selected genes like *SOD1 *and *DYRK1A *[8]. Recent global transcriptome studies with microarrays, however, have generated a more complex picture in the sense that not all HSA21 genes have an elevated expression level as expected [9,10]. An alternative hypothesis is that the phenotype is due to an unstable environment resulting from the dosage imbalance of the hundreds of genes on HSA21 which determines a non-specific disturbance of genomic regulation and expression. The significantly higher inter-individual variability in DS, as compared to euploid, individuals supports this hypothesis [11]. Moreover, the two hypotheses could be coexistent [3]. In both hypotheses it is understood that besides alterations of gene expression of HSA21 genes there are numerous genome-wide effects that lead to the dysregulation of many non-HSA21 genes through molecular pathways and interactions.

Many studies on the transcriptome and proteome levels have been conducted to understand the causal relationship between genes at dosage imbalance and DS phenotypes [12]. Gene expression profiles have been analysed from DS fetal [13] and adult human tissues [6]. Additionally, two classes of mouse models [14] have been developed for investigating the molecular genetics of DS, either mouse models with partial trisomies of the syntenic regions of HSA21 in mouse chromosomes 10, 16 or 17, such as Ts16 [15], Ts65Dn [16] and Ts1Cje mice [17], or transgenic mice for specific genes such as *SOD1 *[18]. Studies of gene expression profiles in human DS samples and mouse models have shown high genome-wide variability [11,19-22]. Furthermore, differences due to the applied experimental platforms, specific tissues, developmental stages or the triplicated segments under study introduce a high variation to the assessment of genome-wide effects of DS. Here, integrative and comparative studies are pivotal for the analysis of the complex nature of gene expression and regulation in DS at a more general level [2,23].

Meta-analysis was proven to be a valid strategy to extract consistent information from heterogeneous data, in particular with respect to complex phenotypes for example cancer [24], Alzheimer [25] and type-2 diabetes mellitus [26]. The purpose of meta-analysis is to compensate experiment-specific variations and to reveal consistent information across a wide range of experiments. To date, such a meta-analysis of DS data is missing.

In this paper we describe a comprehensive meta-analysis from 45 different DS studies on human and mouse on the transcriptome and proteome level including quantitative data such as Affymetrix microarrays, RT-PCR and MALDI studies as well as qualitative data such as SAGE and Western blot analyses. We applied an established computational framework [26] and identified 324 genes with consistent dosage effects in many of these studies. As expected, we observed a high fraction of HSA21 genes (N = 77) but also a large amount of non-HSA21 genes (N = 247). Besides well investigated genes in the context of DS we detected a significant proportion of novel ones (N = 62). The 324 genes were further investigated using functional information, molecular interactions and promoter analysis revealing over-represented motifs of four transcription factors: *RUNX1*, *E2F1*, *STAF/PAX2 *and *STAT3*. In order to test the relevance of the 324 genes for more general brain phenotypes we used independent publicly available data on cerebral pathologies not related to DS and identified a subset of 79 DS genes that were differentially expressed in these studies. The detected dosage effects can be used as a resource for further studies of DS pathology, functional experiments and the development of therapies. All data have been agglomerated and made available through a web server that tracks results of the meta-analysis http://ds-geneminer.molgen.mpg.de/ and that enables the community to validate any gene of interest in the light of the experimental data.

## Results

### Genome-Wide Dosage Effects

Genome-wide dosage effects were computed with the numerical scoring method described in Material and Methods. In total, 45 case-control experiments were interrogated (Additional file 1, Table S1), the alteration for each gene between the trisomic and normal states was scored in each experiment, gene scores were summarised across all experiments and the significance of the summarised scores was judged with a Bootstrap approach. This procedure resulted in a cut-off score value of 3.67 and identified 324 genes as being predominantly affected by DS. The thirty genes with the highest dosage effects, either on HSA21 or on other chromosomes, are listed in Table 1. The entire gene list is given in Additional file 1, Table S2.

**Table 1:** Top thirty DS dosage effects on A) HSA21 and B) other chromosomes

A) Direct effects								
**Ensembl**	**HUGO**	**Score**	**Entropy**	**Chromo-some**	**Start** **position**	**End** **position**	**Band**	**CNV**

ENSG00000154734	ADAMTS1	18.487	4.083	chr21	28208606	28217728	q21.3	
ENSG00000159228	CBR1	17.518	4.509	chr21	37442239	37445464	q22.12	
ENSG00000159140	SON	15.920	4.712	chr21	34914924	34949812	q22.11	
ENSG00000142168	SOD1	15.817	4.372	chr21	33031935	33041244	q22.11	
ENSG00000182670	TTC3L, TTC3	15.637	4.542	chr21	38445526	38575413	q22.13	
ENSG00000142192	APP	15.489	4.412	chr21	27252861	27543446	q21.3	
ENSG00000159128	IFNGR2	15.006	4.640	chr21	34757299	34851655	q22.11	
ENSG00000182240	BACE2	14.156	4.140	chr21	42539728	42648524	q22.2	
ENSG00000156256	USP16	13.713	4.378	chr21	30396950	30426809	q21.3	
ENSG00000159131	GART	13.570	4.564	chr21	34870940	34915797	q22.11	
ENSG00000157540	DYRK1A	13.163	4.405	chr21	38739236	38887680	q22.13	
ENSG00000157557	ETS2	13.088	4.440	chr21	40177231	40196879	q22.2	
ENSG00000159231	CBR3	12.185	3.954	chr21	37507210	37518864	q22.12	YES
ENSG00000159082	SYNJ1	11.880	4.284	chr21	33997269	34100359	q22.11	
ENSG00000142188	TMEM50B	11.516	3.803	chr21	34804792	34853499	q22.11	YES
ENSG00000159110	IFNAR2	11.280	4.360	chr21	34602206	34656082	q22.11	
ENSG00000157538	DSCR3	11.254	4.316	chr21	38591910	38640262	q22.13	
ENSG00000157601	MX1	10.976	3.545	chr21	42792231	42831141	q22.3	
ENSG00000159267	HLCS	10.764	4.183	chr21	38123493	38362536	q22.13	YES
ENSG00000159200	RCAN1	10.719	3.356	chr21	35885440	35987441	q22.12	
ENSG00000159147	DONSON	10.435	4.295	chr21	34947783	34961014	q22.11	
ENSG00000156261	CCT8	10.361	4.560	chr21	30428126	30446118	q21.3	
ENSG00000183486	MX2	10.179	3.598	chr21	42733870	42781317	q22.3	
ENSG00000154727	GABPA	9.936	4.032	chr21	27106881	27144771	q21.3	
ENSG00000160200	CBS	9.284	3.907	chr21	44473301	44497053	q22.3	
ENSG00000159216	RUNX1	9.129	3.783	chr21	36160098	37357047	q22.12	
ENSG00000183527	PSMG1	8.903	3.733	chr21	40546695	40555777	q22.2	
ENSG00000182093	WRB	8.837	4.149	chr21	40752170	40800454	q22.2	
ENSG00000154736	ADAMTS5	8.746	4.221	chr21	28290231	28338832	q21.3	
ENSG00000159197	KCNE2	8.654	3.660	chr21	35736323	35743440	q22.11	YES

**B) Indirect effects**								

**Ensembl**	**HUGO**	**Score**	**Entropy**	**Chromo-some**	**Start** **position**	**End** **position**	**Band**	**CNV**

ENSG00000117289	TXNIP	8.790	3.281	chr1	145438469	145442635	q21.1	YES
ENSG00000133110	POSTN	8.301	2.437	chr13	38136722	38172981	q13.3	YES
ENSG00000118785	SPP1	7.232	3.159	chr4	88896802	88904563	q22.1	
ENSG00000113140	SPARC	7.035	3.338	chr5	151040657	151066726	q33.1	
ENSG00000125968	ID1	6.987	3.164	chr20	30193086	30194318	q11.21	
ENSG00000136235	GPNMB	6.943	2.047	chr7	23275586	23314727	p15.3	
ENSG00000171951	SCG2	6.747	2.950	chr2	224461658	224467221	q36.1	
ENSG00000135821	GLUL	6.604	3.702	chr1	182350839	182361341	q25.3	
ENSG00000123610	TNFAIP6	6.575	2.377	chr2	152214106	152236560	q23.3	
ENSG00000118523	CTGF	6.567	2.996	chr6	132269316	132272513	q23.2	
ENSG00000168209	DDIT4	6.477	3.318	chr10	74033678	74035794	q22.1	
ENSG00000162407	PPAP2B	6.350	3.343	chr1	56960419	57110974	p32.2	
ENSG00000038427	VCAN	6.240	2.958	chr5	82767284	82878122	q14.2	
ENSG00000151491	EPS8	6.194	3.143	chr12	15773076	15942510	p12.3	
ENSG00000189067	LITAF	6.185	3.255	chr16	11641582	11680806	p13.13	
ENSG00000164692	COL1A2	6.148	2.852	chr7	94023873	94060544	q21.3	
ENSG00000204388	HSPA1B	6.109	2.550	chr6	31795688	31798031	p21.33	
ENSG00000162692	VCAM1	6.012	2.533	chr1	101185305	101204601	p21.2	
ENSG00000154096	THY1	5.974	3.244	chr11	119288888	119293854	q23.3	
ENSG00000135919	SERPINE2	5.904	3.048	chr2	224839765	224904036	q36.1	
ENSG00000172201	ID4	5.887	3.037	chr6	19837617	19840915	p22.3	
ENSG00000114315	HES1	5.884	2.874	chr3	193853934	193856521	q29	
ENSG00000172893	DHCR7	5.883	3.441	chr11	71145457	71159477	q13.4	
ENSG00000204262	COL5A2	5.857	3.174	chr2	189896622	190044605	q32.2	
ENSG00000149257	SERPINH1	5.846	3.146	chr11	75273170	75283844	q13.5	
ENSG00000176697	BDNF	5.805	2.416	chr11	27676440	27743605	p14.1	
ENSG00000182551	ADI1	5.782	3.081	chr2	3501693	3523507	p25.3	
ENSG00000079739	PGM1	5.661	3.251	chr1	64058947	64125916	p31.3	
ENSG00000108821	COL1A1	5.527	3.004	chr17	48260650	48278993	q21.33	
ENSG00000187498	COL4A1	5.514	3.396	chr13	110801318	110959496	q34	

**Figure 1:**
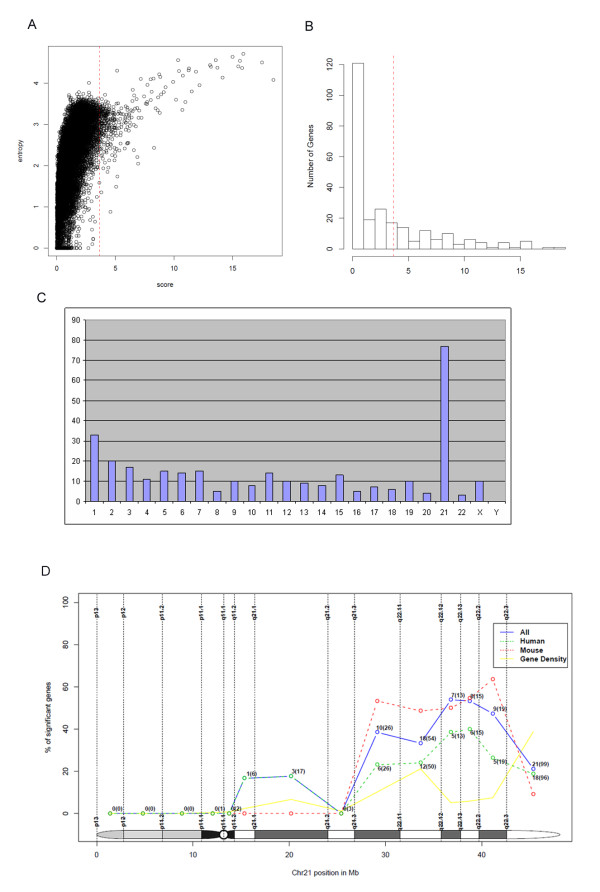
**Characterization of dosage effects**. A) Entropy (Y-axis) vs. score of dosage effect (X-axis) for all genes, B) Histogram of scores for all 255 HSA21 genes accessible with the experiments under study, C) Distribution of genomic locations of the 324 candidate genes, D) Cytogenetic location of 77 HSA21 genes that show significant dosage effects for all experiments (blue line). Additionally, the same meta-analysis approach has been conducted with human (green line) and mouse (red line) data separately. The yellow line plots the relative number of HSA21 genes within each band (gene density). Y-axis shows percentage of significant genes with respect to all genes annotated for the chromosomal band.

The meta-analysis identified genes that showed consistent changes in many of the different experiments rather than genes that were affected by a single (or few) experiment(s) (Figure 1A). This is an important fact since, for example, different mouse models have different coverage of triplicated HSA21 genes, and, thus, might introduce model-specific bias [14]. The consistency of the dosage effect was measured for each gene with an entropy criterion (see Materials and Methods) and Figure 1A reveals a strong preference for the selection of high-entropy genes. Highest scores were assigned to HSA21 genes (Figure 1B) what indicates that the meta-analysis scores reflect the effect of an extra chromosome 21 on gene expression (Table 1). While proportionally most dosage effects were identified for HSA21 genes (77 out of 324), the majority of genes (247 out of 324) was located on other chromosomes highlighting the genome-wide impact of DS (Figure 1C).

Genome-wide dosage effects underlined the severe phenotypic consequences of DS caused by genes with a major role in human development (Additional file 2, Figure S1). Of the 247 non-HSA21 genes, 72 were associated with development, in particular with respect to organ development (62 genes, GO:0048513), tissue development (34 genes GO:0009888) and cell development (30 genes, GO:0048468). Amongst these genes were known interactors of HSA21 genes, for example *REST *(RE1-silencing transcription factor). *REST *modulates expression of genes encoding fundamental neuronal functions including ion channels, synaptic proteins and neurotransmitter receptors and has been linked to an inherited form of mental retardation. Recently, Canzonetta et al. [5] demonstrated that the region capable of affecting *REST *levels, in both mouse and human cells, could be assigned to the *DYRK1A *locus on HSA21 which was found among the top-scoring HSA21 genes (Table 1).

*TXNIP *(thioredoxin interacting protein) had the highest dosage effect (8.79) of all non-HSA21 genes. It has weak association with DS yet (through *S100B *[27]) but could play a major role for several DS phenotypes. It is a key signalling molecule involved in glucose homeostasis [28], cardiovascular homeostasis [29] and leukaemia [30].

Enrichment of genomic location with respect to the 324 genes was observed in regions of HSA21 and the respective syntenic regions on mouse chromosomes 16, 17 and 10 (Additional file 3, Figure S2). Moreover, in the human genome, additional enrichment on chr3q24 was computed containing the genes *GYG1 *(glicogenin), *PLOD2 *(involved in bone morphogenesis), *PLSCR4 *and *CHST2 *(involved in inflammatory response in vascular endothelial cells).

### Dosage Effects on HSA21

Proportionally HSA21 contributed mostly to the detected dosage effects (Figure 1C). On the other hand, it is remarkable that only a third of all HSA21 genes (77 out of 255 studied here using the Ensembl genome annotation [31]) showed consistent effects across the different experiments (see also Discussion). While 57 genes had a positive score below the significance threshold of 3.67 indicating relevance with respect to specific experiments only, 121 genes had a score near zero indicating that dosage effects were either compensated or not detected with the selected experimental data (Figure 1B).

HSA21 dosage effects included, for example *APP *(beta-amyloid precursor protein) involved in senile plaque formation in DS and Alzheimer's disease [3], *SOD1 *(superoxide dismutase 1), a key enzyme in the metabolism of oxygen-derived free radicals [3], *DYRK1A *(dual-specificity tyrosine-(Y)-phosphorylation regulated kinase 1A) involved in neuroblast proliferation, crucial for brain function, learning and memory [32], *RUNX1 *(runt-related transcription factor 1) which plays a critical role in normal hematopoiesis [33], or *GABPA *(GA binding protein transcription factor, alpha subunit 60 kDa) encoding a DNA binding domain with a huge variety of targets including genes from different cell/tissue specificities and functions [34]. HSA21 genes were mostly up-regulated in gene expression studies (69 out of 77) with the exception of eight genes that were either variable or down-regulated (*SLC5A3*, *MRPS6*, *B3GALT6*, *CBS*, *KCNJ6*, *KCNJ15*, *CLDN14*, *COL18A1*). Possible explanations for this observation might be tissue-specificity of gene expression as in the case of *MRPS6 *which was mostly up-regulated in brain samples and down-regulated in other tissues like heart or kidney, or differences in human and mouse gene expression as in the case of *CBS *which was up-regulated in human but down-regulated in mouse experiments what might be caused by differential tissue specificity of the orthologous mouse gene [35].

Three genomic regions on HSA21 were enriched with the significant genes using the MSigDB_c1 positional database: chr21q22, chr21q21 and chr21q11, located on the q-terminal arm (Figure 1D). This contradicts the hypothesis that a single region on HSA21 could be responsible for the molecular and phenotypic consequences of DS with only a few responsive genes [36,37]. Rather our findings support studies that identified more than one HSA21 region causative for DS phenotypes so that the dosage effects were not uniformly distributed along the chromosome but rather enriched in certain regions on HSA21 similar to the results in [38,39].

### Functional Annotation Using Gene Enrichment Analysis

**Figure 2:**
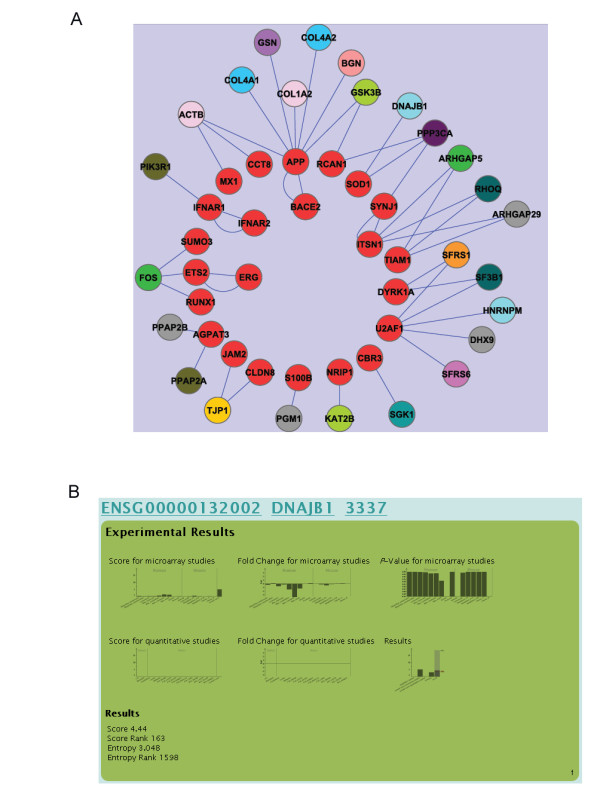
**Molecular interactions of HSA21 genes**. A) Interactions of HSA21 genes (red) with non-HSA21 genes (other colours). Same colours of the gene nodes refer to the same chromosome. B) Example of consistent down-regulation of *DNAJB1 *as a consequence of HSA21 imbalance visualized in the web browser.

Functional annotation of biological pathways was retrieved from the ConsensusPathDB [40], a meta-database that summarizes the content of 22 human interaction databases. A total of 1,695 pre-defined pathways were screened with the 324 genes using gene set enrichment analysis [41]. A total of 277 pathways were found significantly enriched (family-wise error rate (FWER)<0.01) of which several pathways were associated with neurological and neuropathological processes (Table 2). These pathways referred mainly to *(i) *neurodegeneration (e.g. *Huntington's disease, Alzheimer's disease or Parkinson's disease*) and *(ii) *defects in synapsis (e.g. *Axon guidance*, *NGF signaling*). Furthermore, the results emphasized the role of tyrosine-kinase receptors in DS pathology (for example *P75(NTR)- mediating signalling or NGF signalling via TRKA*) which interact directly with *BDNF *(brain-derived neurotrophic factor). Moreover, our results showed gene dosage effects caused either directly by genes located on HSA21 (e.g. *SOD1*, *APP*, *DONSON*, *TIAM1*, *COL6A2*, *ITSN1 *and *BACE2*) or indirectly by HSA21 interactors, highlighting the intrinsic complexity of the DS pathology. For example, *PIK3R1 *de-regulation impacts on many of these pathways and is a direct interactor of *IFNAR1*, a significant DS gene. A similar effect can be observed for *TPJ1A *that has interactions with HSA21 genes *JAM2 *and *CDLN8 *both showing consistent dosage effects (cf. Figure 2A).

**Table 2:** Enriched neuropathological pathways.

PATHWAY (Source Database)	Pathway size	P-value	FWER P-value	Genes on HSA21	HSA21 Interactors	Others
HUNTINGTONS DISEASE (KEGG)	159	0	0	SOD1; DONSON	REST	BDNF; SOD2
ALZHEIMERS DISEASE (KEGG)	147	0	0	APP; BACE2; DONSON	PPP3CA; GSK3B	CAPN2
SIGNALLING BY NGF (REACTOME)	209	0	0	ITSN1; TIAM1	PIK3R1; GSK3B	RPS6KA2; RAP1A; KRAS
AXON GUIDANCE (REACTOME)	256	0	0	COL6A2	GSK3B;COL1A1; COL1A2; COL4A1; COL4A2	COL5A2; DPYSL3; RPS6KA2; LAMB1; COL3A1; COL5A1; ALCAM; KRAS
PARKINSONS DISEASE (KEGG)	105	0	0	DONSON		UBE2G2
P75(NTR)-MEDIATED SIGNALING (PID)	68	0	0	APP	PIK3R1	BDNF
NOTCH (NETPATH)	61	0	0	APP	PIK3R1; GSK3B	
NEUROTROPHIN SIGNALING PATHWAY (KEGG)	121	0	0		PIK3R1; GSK3B	BDNF; RPS6KA2; RAP1A; KRAS
NGF SIGNALLING VIA TRKA FROM THE PLASMA MEMBRANE (REACTOME)	127	0	0		PIK3R1; GSK3B	RPS6KA2; RAP1A; KRAS
MEMBRANE TRAFFICKING (REACTOME)	87	0	0		TJP1	GJA1; COPG
NEUROTROPHIC FACTOR-MEDIATED TRK RECEPTOR SIGNALING (PID)	60	0	0	TIAM1	PIK3R1	BDNF; RAP1A; KRAS
EPO SIGNALING (INOH)	180	0	0		PIK3R1; GSK3B	
CDC42 SIGNALING EVENTS (PID)	68	0	0	TIAM1	PIK3R1; GSK3B	EPS8; YES1
L1CAM INTERACTIONS (REACTOME)	93	0	0			LAMB1; ALCAM1; RPS6KA2

### Dosage Effects on Transcriptional Regulation

Dysregulation of transcriptional regulation is widely reported in DS [34]. Among the 324 significant genes were 13 transcription factors (TFs) (*PSIP1, RBPJ, TCF4, HES1, ETS2, BACH1, RUNX1, GABPA, SNAI2, REST, LITAF, EGR1, FOS*), 6 TFs (*PSIP1, HOXC8, DLX5, HIVEP3, ZNF187, ATF6*) had significant enrichment of their targets as retrieved by the TRANSFAC [42] database. Additionally, 57 TFs had significant enrichment of their interacting proteins when judged with physical interactions retrieved from the ConsensusPathDB [40]. In total, 70 different TFs were identified as being (directly or indirectly) affected by dosage imbalances. The list of TFs and their associated functional categories is given in Additional file 1, Table S3. GO categories indicate a broad impact of transcriptional regulation for neurological development, the central nervous system development (*RUNX1 *and *TP53*), nervous system development (*DLX5, FOS, HES1, STAT3 *and *EP300*), axonogenesis (*DLX5*, *NOTCH1 *and *CREB1*), neuron differentiation (*HOXC8*, *NOTCH1 *and *RUNX1*), negative regulation of neuron differentiation (*HES1*, *NOTCH1 *and *REST*) and regulation of long-term neuronal synaptic plasticity and learning or memory (*EGR1 *and *JUN*). Other prominent categories refer to organ development (*RBPJ, ETS2, GABPA *and *SNAI2*) and stress response (*ATF6*, *FOS *and *RELA*).

We further analyzed the promoter sequences of the 324 genes for enrichment of transcription factor binding sites using the AMADEUS software [43]. Significant enrichment was computed for 4 TF motifs, *E2F1, RUNX1, STAF/PAX2 *and *STAT3 *(Table 3). Enrichment was evident for *RUNX1*, which is among the most studied genes implicated in DS. The implication of *E2F1 *in DS was also previously reported [34] and could be responsible for impaired cell proliferation documented for hippocampus, cerebellum and astrocytes of DS mouse models.

**Table 3:** Enriched TFBSs.

TF	Description	Cromo-some	P-Value	Binding motif	Strand
E2F1	E2F transcription factor 1	chr20	9.3*10^-18^	[C/t][C/a][G/c]C[c/a][C/g][G/c][C/T][G/c]A	-
RUNX1	runt-related transcription factor 1	chr21	4.0*10^-18^	[C/a/t][T/a/g][G/C]{A}[G/c]{A}T[C/A][G][C/a/t/g]	+
STAF/ PAX2	paired box 2	chr10	8.4*10^-18^	[A/g][A/g]A[C/T/a][T/g/a][T/c][C/t][C/g][C/a]	+
STAT3	signal transducer and activator of transcription 3 (acute-phase response factor)	chr17	8.4*10^-17^	GAA[A/T][C/T]G[C/T][C/g/t][A/T][C/T/g]	+

Binding motifs have been represented using the IUPAC nomenclature and incorporating lower case for low frequency bases.

### Dosage Effects and Molecular Interactions

Molecular interactions among the 324 significant genes on HSA21 and on other chromosomes exhibited a complex network supporting the important role of physical interactions as transmitter of dosage effects (Figure 2A). The consequences of HSA21 triplication on the interacting genes was fairly stable as Figure 2B demonstrates. For example, *DNAJB1 *(DnaJ (Hsp40) homolog, subfamily B, member 1) and *PPP3CA *(protein phosphatase 3, catalytic subunit, alpha isozyme, data not shown), both interacting with *SOD1*, were consistently and significantly down-regulated in the human microarray experiments as the fold-changes and P-values indicate. Opposite trends were observed for *TJP1 *and *RHOQ*.

### Assessing General Relevance of DS Dosage Effects for Neurological Processes

**Figure 3:**
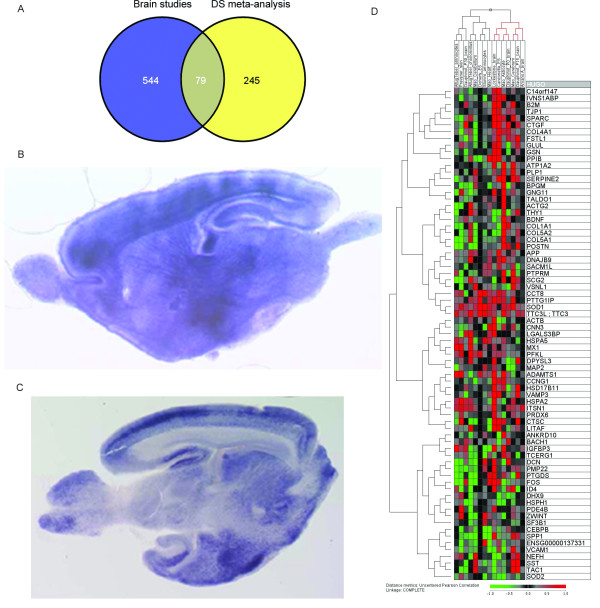
**Brain-related dosage effects**. A) Venn diagram showing the overlap of the 324 significant genes with 623 genes identified by independent mouse studies related to brain phenotypes; B) RNA in situ hybridisations of *BACH1 *in postnatal mouse embryonic brain slices. C) In situ hybridisation of *TTC3 *in the same tissue. Images kindly provided by the HSA21 consortium ([4]; http://chr21.molgen.mpg.de/hsa21). D) Hierarchical clustering of 79 genes related to non-DS general brain disorders with the DS gene expression data sets. Clustering was performed with the J-Express 2009 software using Pearson correlation as similarity measure and complete linkage as update rule.

We were further interested in identifying, among the 324 genes, those which were relevant for other brain disorders. To achieve this, we interrogated 19 independent data sets derived from publicly available microarray data (Additional file 1, Table S4). These studies followed heterogeneous research questions on different cerebral pathologies and identified a total of 623 differentially expressed genes. Gene set enrichment analyses [41] with the 324 genes and the corresponding lists of differentially expressed genes were significant for 10 of these 19 studies with 79 overlapping genes (Figure 3A). Furthermore, we used the HSA21 database http://chr21.molgen.mpg.de/hsa21[4], a resource of RNA in situ hybridizations in postnatal mouse brain sections, in order to provide independent supporting evidence of brain expression of these 79 genes as shown for example for *BACH1 *(basic leucine zipper transcription factor 1) and *TTC3 *(tetratricopeptide repeat domain 3) (Figure 3B and 3C).

Additionally, we investigated the expression patterns of the 79 genes across the DS microarray experiments used for this meta-analysis and could identify brain-related signatures, for example, a clear up-regulation in brain tissues for the cluster containing *C14orf147, IVSNS1ABP, B2M, TPJ1, SPARC, CTGF, COL4A1 *and *FSTL1 *(Figure 3D).

### Novel Dosage Effects

**Figure 4:**
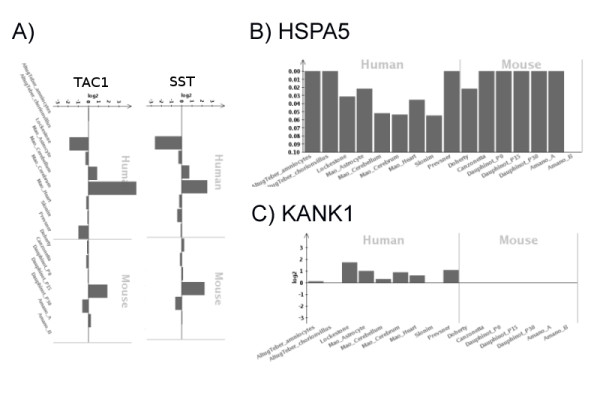
**Novel DS dosage effects visualised with the web browser**. A) *SST *and *TAC1 *have been previously reported as acting in a complex. The deregulated profile of these genes correlates was shown here with the fold-change view of the web browser. B) *HSPA5 *is a novel gene for DS implicated in neurodegeneration which is also a target of the *ATF6 *TF whose target set was enriched with significant genes. The histogram displays the p-values for this gene in individual studies. C) *KANK1*, a gene previously related with paternally inherited cerebral palsy, shows a consistent trend of up-regulation in the considered studies as shown with the fold-change view of the web browser.

To identify DS-relevant "novel" dosage effects we excluded from the 324 genes *(i) *HSA21 genes, *(ii) *genes that interacted with HSA21 genes, as well as *(iii) *genes that were associated with DS in the literature (Table 4). Remaining candidates (N = 62) comprised BDNF-related genes (*SST*), MAPK-pathway genes (*KRAS*, *IGF1R*, *GNG11 *and *RAP1A*), genes related with leukemia (*SFRP1*) and Rho-Proteins (*DHCR7 *and *RAB21*). *SST *was found as co-expressed in previous studies with *TAC1 *[44] which is also significant in our meta-analysis and both showed a strong correlation across DS studies (Figure 4A).

**Table 4:** Novel DS dosage effects

Ensembl	HUGO	Score	Entropy	Chromo-some	Start position	End position	Band	CNV
ENSG00000133110	POSTN	8.301	2.437	chr13	38136722	38172981	q13.3	YES
ENSG00000135919	SERPINE2	5.904	3.048	chr2	224839765	224904036	q36.1	
ENSG00000172893	DHCR7	5.883	3.441	chr11	71145457	71159477	q13.4	
ENSG00000135744	AGT	5.467	2.799	chr1	230838269	230850043	q42.2	
ENSG00000159176	CSRP1	5.424	3.136	chr1	201452658	201478584	q32.1	
ENSG00000178695	KCTD12	5.344	2.373	chr13	77454312	77460540	q22.3	
ENSG00000183087	GAS6	5.129	2.904	chr13	114523524	114567046	q34	
ENSG00000164106	SCRG1	5.127	2.728	chr4	174309300	174320617	q34.1	
ENSG00000166923	GREM1	5.073	1.486	chr15	33010175	33026870	q13.3	YES
ENSG00000163754	GYG1	4.933	3.129	chr3	148709128	148745419	q24	
ENSG00000155380	SLC16A1	4.878	2.927	chr1	113454469	113499635	p13.2	YES
ENSG00000166033	HTRA1	4.811	3.101	chr10	124221041	124274424	q26.13	YES
ENSG00000145632	PLK2	4.785	2.811	chr5	57749809	57756087	q11.2	
ENSG00000115380	EFEMP1	4.726	2.257	chr2	56093102	56151274	p16.1	
ENSG00000060237	WNK1	4.637	2.765	chr12	862089	1020618	p13.33	YES
ENSG00000103888	KIAA1199	4.581	0.885	chr15	81071684	81244117	q25.1	
ENSG00000113810	SMC4	4.462	3.372	chr3	160117062	160152750	q25.33	
ENSG00000198356	ASNA1	4.431	3.067	chr19	12848306	12859137	p13.2	
ENSG00000122952	ZWINT	4.415	3.266	chr10	58116989	58121036	q21.1	
ENSG00000157005	SST	4.401	1.954	chr3	187386694	187388187	q27.3	
ENSG00000117519	CNN3	4.384	3.253	chr1	95362507	95392834	p21.3	
ENSG00000107104	KANK1	4.352	2.508	chr9	470291	746105	p24.3	YES
ENSG00000151414	NEK7	4.329	1.848	chr1	198126121	198291550	q31.3	
ENSG00000044574	HSPA5	4.261	3.449	chr9	127997132	128003609	q33.3	
ENSG00000128590	DNAJB9	4.251	3.241	chr7	108210012	108215294	q31.1	
ENSG00000127920	GNG11	4.226	2.747	chr7	93551011	93555831	q21.3	
ENSG00000008083	JARID2	4.161	3.203	chr6	15246527	15522253	p22.3	
ENSG00000119938	PPP1R3C	4.159	3.036	chr10	93388199	93392858	q23.32	
ENSG00000049245	VAMP3	4.146	3.036	chr1	7831329	7841492	p36.23	
ENSG00000120694	HSPH1	4.129	3.310	chr13	31710762	31736502	q12.3	
ENSG00000168214	RBPJ	4.127	3.291	chr4	26321332	26436753	p15.2	
ENSG00000162909	CAPN2	4.111	3.020	chr1	223889347	223963720	q41	YES
ENSG00000166147	FBN1	4.106	2.070	chr15	48700505	48937918	q21.1	YES
ENSG00000100941	PNN	4.081	3.380	chr14	39644425	39652422	q21.1	
ENSG00000132640	BTBD3	4.074	3.478	chr20	11871371	11907257	p12.2	YES
ENSG00000128708	HAT1	4.064	3.158	chr2	172778958	172848599	q31.1	YES
ENSG00000176105	YES1	4.047	2.855	chr18	721588	812327	p11.32	
ENSG00000152377	SPOCK1	4.025	3.083	chr5	136310987	136835037	q31.2	
ENSG00000136026	CKAP4	4.018	2.754	chr12	106631659	106641908	q23.3	
ENSG00000198121	LPAR1	3.979	2.858	chr9	113635543	113800738	q31.3	
ENSG00000140443	IGF1R	3.951	3.376	chr15	99192200	99507759	q26.3	
ENSG00000198730	CTR9	3.891	3.310	chr11	10772803	10801287	p15.3	
ENSG00000162616	DNAJB4	3.869	3.035	chr1	78444859	78483648	p31.1	
ENSG00000104332	SFRP1	3.825	2.587	chr8	41119483	41166992	p11.21	
ENSG00000116473	RAP1A	3.824	2.769	chr1	112084840	112259313	p13.2	
ENSG00000172500	FIBP	3.804	3.309	chr11	65651211	65656010	q13.1	YES
ENSG00000133703	KRAS	3.801	3.338	chr12	25358182	25403854	p12.1	
ENSG00000163032	VSNL1	3.798	3.099	chr2	17720393	17838285	p24.2	
ENSG00000134684	YARS	3.765	3.431	chr1	33240840	33283754	p35.1	
ENSG00000105854	PON2	3.764	2.862	chr7	95034179	95064510	q21.3	
ENSG00000148943	LIN7C	3.763	3.033	chr11	27516124	27528303	p14.1	
ENSG00000162734	PEA15	3.747	3.418	chr1	160175127	160185166	q23.2	
ENSG00000103187	COTL1	3.731	3.304	chr16	84599200	84651683	q24.1	YES
ENSG00000198648	STK39	3.722	3.439	chr2	168810530	169104651	q24.3	
ENSG00000100577	GSTZ1	3.713	2.759	chr14	77787230	77797939	q24.3	
ENSG00000080371	RAB21	3.707	3.312	chr12	72148658	72181150	q21.1	YES
ENSG00000136108	CKAP2	3.688	2.960	chr13	53029495	53050485	q14.3	
ENSG00000066583	ISOC1	3.686	2.655	chr5	128430442	128449721	q23.3	
ENSG00000143420	ENSA	3.681	3.276	chr1	150573327	150602098	q21.3	
ENSG00000114353	GNAI2	3.680	3.138	chr3	50263724	50296787	p21.31	YES
ENSG00000140105	WARS	3.671	2.994	chr14	100800125	100842680	q32.2	
ENSG00000018625	ATP1A2	3.670	2.733	chr1	160085549	160113381	q23.2	

Novel candidates are associated with neurodegenerative disorders including Alzheimer's disease (*VSNL1*), prion disease (*SCRG1, HSPH1, HSPA5 *(Figure 4B) and *CTR9*) and age-related degeneration (*GAS6 *and *GNG11*). Moreover, candidates could explain evident DS features (Additional file 1, Table S5): *(i) *genes related to neurogenesis and neurite outgrowth (*LPAR1 *[45], *LIN7C*, *JARID2*, *GREM1, SERPINE2, IGFR1 *and *SPOCK1*) that could be related with mental retardation or cognitive impairment, *(ii) *genes involved in synapsis (*AGT, KRAS, ATP1A2, GNAI2, SST *and *LIN7C*) *(iii) *cytoskeletal related proteins (*KANK1 *[46]; Figure 4C), *CKAP2, CKAP4, HAT1, NEK7 *and *VAMP3*), *(iv) *macular degeneration genes [47] or genes (*HTRA1 *and *EFEMP1*) associated with age-related visual problems [48], *(v) *genes *(AGT, CNN3, FBN1, RBPJ, PON2, POSTN, RAP1A, WNK1 *and *STK39*) that were related with cardiac impairments and could be candidates to explain this DS characteristic [49], and *(vi) *genes related with cancer (*BTBD3 *[50], *DNAJB4 *[51], *FIBP *[52] and *GSTZ1 *[53]) [54].

These examples show that the meta-analysis approach identified multiple additional genes that might be involved in DS pathology. In order to enable the community to check any particular gene of interest for DS relevance in the studies under analysis, we have agglomerated all information of the meta-analysis into a WEB-interface http://ds-geneminer.molgen.mpg.de/. Examples of possible views and information are shown in Figure 4.

## Discussion

The statistical meta-analysis approach was described previously by Rasche et al. [26]. The score computed with meta-analysis correlates with entropy (Figure 1A) indicating the ability to identify general dosage effects across many experiments that might be of more phenotypic relevance than very specific ones. Additional file 4, Figures S3A and B provide an overview of the different sources of data, including two organisms (human and mouse), different tissues (brain, heart and others), different stages of development (adult, postnatal, embryonic) and different mouse models (Ts65DN, Ts1Cje, Tc1). It is *per se *interesting that, in spite of such heterogeneity, common dosage effects could be identified at all and it should be highlighted that whole-genome data was fairly robust across experiments. Additional file 4, Figure S3D shows the overall correlation of the quantitative values of PCR and microarray values averaged from all experiments with only few genes in the non-concordant sectors of the graph (red points).

The score used in this analysis allows detecting genes that could be either up- or down-regulated in different studies. An overview of the fold-changes for the genes across the different experiments is given in Additional file 1, Table S6. Because genes might change their expression level depending on the developmental state, tissue or because of other variables, we expected that this flexibility allows checking the hypothesis of random disturbances as well as the hypothesis of increased expression of HSA21 genes. We detected a clear enrichment of up-regulated genes on the q-terminal part of HSA21 (Figure 1D and Additional file 3, Figure S2). However, not a single region was identified but rather several smaller regions on HSA21 that agglomerate a large amount of significant dosage effects. This finding was also elaborated before (Korbel et al. [38] and Lyle R et al. [39]) using two independent data sets to characterize the molecular HSA21 regions in a set of DS-patients with partial duplications.

We studied 255 HSA21 genes matched with the probe sets from the microarrays. Of these only 77 showed consistent dosage effects (Figure 1). While 165 HSA21 genes had score values different from zero indicating response in some of the microarray studies, 90 HSA21 genes were not responsive at all and provide evidence for a strong mechanism of dosage compensation. On the other hand, these figures could also reflect the limitation of detecting reliable fold-changes of low magnitude with microarray technology. Furthermore, experiments covered only a limited amount of tissues so that it is likely that some genes were missed simply because they were not responsive in the tissues under analysis. However, having brain as the dominant whole-genome sample source this should ensure expression of most of the genes. Microarray data was focused on the Affymetrix platform in order to reduce variance arising from platform inconsistencies. We have also compared our results with additional studies including own previous research [9] and others [55] and found relevance of selected dosage effects with respect to other tissues as well (data not shown). Additional cross-validation was performed with an independent microarray data set [10]. These authors compared human lymphoblastoid cell lines derived from DS patients and normal controls with a custom-made HSA21 array. Yahya-Graison et al. [10] divided the expression ratios in four classes: class I and class II genes were significantly up-regulated, while class III and class IV genes were either compensated or showed variable response. Our meta-analysis revealed a high-degree of concordance taking into account that the cell model, platform and the methodology used were completely different. The meta-analysis scores were significantly higher for class I and II genes than for class III and IV genes (P-value <0.01, Additional file 5, Figure S4). 25 out of 39 class I-II genes revealed a significant score in our meta-analysis (75%).

In this study we monitored molecular interactions of HSA21 genes that might function as drivers of dosage effects (Figure 2A). For example, *(i) TJP1 *(Tight junction protein ZO-1) interacts with two HSA21 genes, *JAM2 *and *CLDN8*, *(ii) FOS *(FBJ murine osteosarcoma viral oncogene homolog) interacts with HSA21 genes *ETS2*, *SUMO3*, *RUNX1 *and indirectly with *ERG*, *(iii) RHOQ *(ras homolog gene family, member Q) interacts directly with *ITSN1 *and *TIAM1 *and indirectly with *SYNJ*, and *(iv) PIK3R1 *interacts directly with *IFNAR1 *and indirectly with *IFNAR2*. It should be emphasized that current information on molecular interactions is far from complete, thus we either might miss important interactions not yet detected and/or we might count false positive interactions due to the high error rates of current annotations of interactions.

Several of the DS genes (N = 79) extrapolated to more general neurological phenotypes (Figure 3A). The dendrogram (Figure 3D) shows further interesting profiles of these genes in the DS samples under analysis: *(i) *differential gene expression in the cerebellum region versus whole "brain" or cerebrum areas which has been reported in other studies (e.g. Moldrich et al. [56]), *(ii) *different patterns of gene expression associated to particular developmental stages (P0, P15 and P30); these changes were reported before by Dauphinot el al. [57], and *(iii) *differences in ES studies.

We further analyzed human and mouse studies separately and found 182 significant dosage effects using only human and 107 dosage effects using only mouse data. The Venn diagram in Additional file 4, Figure S3C clearly shows the benefit in detecting additional dosage effects when mixing the two species. Overlapping dosage effects were detected for 29 genes with both analyses (Additional file 1, Table S7). Results for the human and mouse specific analyses can be found in Additional file 1, Tables S8 and S9. It should be noted here that comparisons between human and mouse using microarrays are inherently difficult and have limitations since the probes for the orthologous mouse and human genes do not correspond well. Furthermore, gene expression variation is generally higher in human individuals compared to mouse inbred strains. Nonetheless, the 107 genes found in the analysis of mouse data (derived from the different mouse models for trisomy 21) represent a core set of genes responsive across different DS mouse models and, thus, could be highly relevant for DS pathogenesis.

In addition to genes commonly related to DS, we have identified novel genes that can be associated with DS phenotypes, in particular with neural development and neurodegeneration. To our best knowledge, this study is the first meta-analysis of genome-wide transcript levels along with other data domains in DS research. The agglomerated data can be accessed through the WEB server at http://ds-geneminer.molgen.mpg.de and the identified dosage effects are a resource for further functional testing and therapeutic development.

## Conclusions

We have identified a set of 324 genes with consistent dosage effects from 45 different experiments related to DS. Since the meta-analysis was enriched with brain experiments, we were able to detect a high fraction of genes related to neuro-development, synapsis and neuro-degeneration. Moreover, our results give more information about known and new pathways related to DS and also about 62 novel candidates. The results of the meta-analysis as well as the source data have been made accessible for the community through a WEB interface.

## Material and methods

### Selection and integration of DS resources

Data sets were selected from heterogeneous technical platforms, different model systems (human cell lines, human tissues, mouse models) and different developmental stages (Additional file 1, Table S1). For each gene and for each source we computed a numerical value that measures its dosage effect. Data categories were either qualitative or quantitative. Qualitative data incorporated a total of 30 published manuscripts including reviews and semi-quantitative studies as well as two SAGE studies [21,58] and were summarised to one score point in order to avoid over-scoring. Here, a "1" referred to the case that the gene was found as DS relevant in one (or more) studies. Quantitative data from differential gene expression studies such as Affymetrix microarrays, RT-PCR, MALDI and other quantitatives techniques were evaluated in order to extract comparable information across the different studies. We considered Affymetrix studies that provided the raw data (CEL file level). Raw data were extracted from Gene Omnibus Expression (GEO, [59]), Array Express [60] or were retrieved from the author's web pages (in total 16 data sets including human tissues and four different mouse models (Ts65Dn, Ts1Cje, Tc1 and Ts + HSA21). Furthermore, we incorporated 18 RT-PCR and MALDI data sets for which the authors displayed the information for all genes under study (either significant or not).

### Mapping of gene IDs

A central pre-requisiste of any meta-analysis approach is the consolidation of the different ID types, for example coming from different organisms and from different versions of chips. We used the Ensembl database (version 56) as the backbone annotation for all studies. IDs were mapped to human Ensembl gene IDs. Mapping and merging of the information was done within R and the BioConductor package. In total, information on 19,388 ENSEMBL genes was mapped.

### Mapping SAGE IDs

Differential expressed tags were extracted from additional files of the studies. Identifiers (based on sequences) were cross-tagged with the information displayed in the updating tables (SAGEmap_Hs and SAGEmap_Mn) from the SAGE site ftp://ftp.ncbi.nlm.nih.gov/pub/sage/mappings.

### Transcriptome data pre-processing and normalization

We incorporated only case-control studies in the meta-analysis in order to derive expression fold-changes. Affymetrix gene chip annotations were adapted from the latest genome annotation (version 12). Affymetrix data were normalized with GC RMA. For transcriptome case-control studies three pieces of information were stored for each gene; *(i) *the fold-change (DS vs. controls), *(ii) *the standard error of the fold-change from the replicated experiments in that study and *(iii) *the expression p-value (presence-call) that indicates whether or not the gene is expressed in the target samples under study. For RT-PCR and MALDI experiments we computed the fold-change of the mean expression (DS vs. controls) as well as the reported standard error of the ratio. When mean and standard variation for each group (DS and controls) was provided we calculated the ratios as well as their associated standard errors.

### Scoring DS dosage effects across studies

In order to score the different categories of information such as binary counts and quantitative gene expression values, we summarized the scores of the individual experiments for each category. For microarray studies, the score of the *i*-th gene in the *j*-th study, s_ij_, was computed as described in Rasche et al. [26]:

Here r_ij _is the fold-change, p_ij _is the average detection p-value and e_ij _is the standard error of the ratio derived from the experimental replicates of the study. Thus, the fold-change is weighted with its reproducibility across the experimental replicates and with the likelihood of the gene being expressed in the study's case or control samples.

For RT-PCR and MALDI studies we applied the following equation:

Here r_ij _is the fold-change and e_ij _is the standard error of the ratio.

The total score of the gene was computed as the sum across all individual study scores.

### Sampling for significance

In order to assess the significance of the overall gene scores we generated random scores by re-sampling the scores 50,000 times with replacement within the same study. Using the random distribution as background we assigned as significant those genes that were above the 99.9 percentile of the background distribution.

### Judging consistency of dosage effects

For each gene, entropy of the score distribution was computed in order to quantify the relevance of the gene across many experiments. Let s_ij _be the score of the ith gene in the jth study, then E_i _is a measure for the uniformity of the score distribution over the individual experiments:

High entropy is assigned to a gene if many experiments contribute to the overall score whereas low entropy is assigned if only a few experiments contribute to the overall score.

### Enrichment analysis

Gene Set Enrichment Analysis (GSEA, [41]) of the 324 genes was performed with respect to pre-defined human pathways agglomerated from 22 pathway resources from the ConsensusPathDB ([40], http://cpdb.molgen.mpg.de. Over-representation analysis of TF target sets was performed with Fisher's test based on annotation from TRANSFAC [42]. Motif enrichment analyses were performed using AMADEUS [43] with significant genes as target sets and all the genes considered in the meta-analysis as background set.

### Selection of independent brain experiments

In order to proof general brain relevance of the 324 genes, we collected DS-independent gene expression studies to decipher brain features, performed with Affymetrix technology and, with experiments deposited in GEO or ArrayExpress (Additional file 1, Table S4). Mostly, these experiments were performed in mouse tissues. For each study we collected one or more resulting gene lists that were evaluated using Gene Set Enrichment Analysis (GSEA, [41]) against the complete list of 19,388 genes ranked by score.

## Abbreviations

DS: Down Syndrome; HSA21: human chromosome 21; TF: Transcription Factor; PCR: Polymerase Chain Reaction; RT-PCR: real-time Polymerase Chain Reaction; MALDI: Matrix-Assisted Laser Desorption/Ionization; SAGE: Serial Analysis of Gene Expression; GO: Gene Ontology; ES: Embryonic Stem Cells; ID:Identifier; GEO: Gene Omnibus Expression; GSEA: Gene Set Enrichment Analysis; CNV: Copy Number Variation; TFBS: Transcriptor Factor Binding Site

## Authors' contributions

MV carried out the systematic revisions, collected the data for the meta-analysis and for the related studies. AR wrote the general code for the meta-analysis. AR and MV adjusted the code for DS study. AT created the browser which allows the results visualization, EMD carried out the transcription factor analysis. MV performed the promoter sequences' analysis and the further statistical analysis. RH conceived of the study, and participated in its design and coordination. MV, RH, HL and LAPJ contributed to the data interpretation and wrote the manuscript. All authors read and approved the final manuscript.

## Supplementary Material

Additional file 1 **Supplementary tables**. Table S1. Data sources used for meta-analysis. Table S2. The 324 candidate genes detected in the meta-analysis study. Table S3. Transcription factors and associated GO terms. Table S4. Cross-validation studies. Table S5. Functional annotation of novel candidates. Table S6. Fold-changes and qualitative data. Table S7. Human and mouse data overlap. Table S8. DS genes derived from meta-analysis of human data. Table S9. DS genes derived from meta-analysis of mouse data. Click here for file

Additional file 2 **Figure S1**. Enrichment of GO categories for organ, tissue and cell development with respect to the significant HSA21 genes (red bars), the significant non-HSA21 genes (green bars) and the non-significant genes (blue bars). Click here for file

Additional file 3 **Figure S2**. Genomic location of DS dosage effects in A) human B) mouse. Significant genes are marked in red, non-significant genes in white. Click here for file

Additional file 4 **Figure S3**. A) Categorization of the 35 qualitative studies, B) Categorization of the 34 quantitative studies. C) Venn diagram of dosage effects detected with mouse and human data alone and with the combination of all data, D) correlation between average PCR and microarray values for the detected 324 dosage effects. Click here for file

Additional file 5 **Figure S4**. Cross-validation with DS dosage effects detected with an HSA21 microarray [54]. Box-plots of meta-analysis scores (Y-axis) for class I and II (dosage effects) and class III and IV (compensation and variable expression) genes as judged by the authors. Click here for file

## References

[B1] Patterson D Genetic mechanisms involved in the phenotype of Down syndrome Ment Retard Dev Disabil Res Rev 2007 13 3 199 206 10.1002/mrdd.20162 17910086

[B2] Antonarakis SE Epstein CJ The challenge of Down syndrome Trends Mol Med 2006 12 10 473 479 10.1016/j.molmed.2006.08.005 16935027

[B3] Rachidi M Lopes C Mental retardation in Down syndrome: from gene dosage imbalance to molecular and cellular mechanisms Neurosci Res 2007 59 4 349 369 10.1016/j.neures.2007.08.007 17897742

[B4] Gitton Y Dahmane N Baik S Ruiz i Altaba A Neidhardt L Scholze M Herrmann BG Kahlem P Benkahla A Schrinner S Yildirimman R Herwig R Lehrach H Yaspo ML A gene expression map of human chromosome 21 orthologues in the mouse Nature 2002 420 6915 586 590 10.1038/nature01270 12466855

[B5] Canzonetta C Mulligan C Deutsch S Ruf S O'Doherty A Lyle R Borel C Lin-Marq N Delom F Groet J Schnappauf F De Vita S Averill S Priestley JV Martin JE Shipley J Denyer G Epstein CJ Fillat C Estivill X Tybulewicz VL Fisher EM Antonarakis SE Nizetic D DYRK1A-dosage imbalance perturbs NRSF/REST levels, deregulating pluripotency and embryonic stem cell fate in Down syndrome Am J Hum Genet 2008 83 3 388 400 10.1016/j.ajhg.2008.08.012 18771760 PMC2556438

[B6] Lockstone HE Harris LW Swatton JE Wayland MT Holland AJ Bahn S Gene expression profiling in the adult Down syndrome brain Genomics 2007 90 6 647 660 10.1016/j.ygeno.2007.08.005 17950572

[B7] Antonarakis SE Lyle R Dermitzakis ET Reymond A Deutsch S Chromosome 21 and down syndrome: from genomics to pathophysiology Nat Rev Genet 2004 5 10 725 38 10.1038/nrg1448 15510164

[B8] Wiseman FK Alford KA Tybulewicz VL Fisher EM Down syndrome--recent progress and future prospects Hum Mol Genet 2009 18 R1 R75 83 10.1093/hmg/ddp010 19297404 PMC2657943

[B9] Kahlem P Sultan M Herwig R Steinfath M Balzereit D Eppens B Saran NG Pletcher MT South ST Stetten G Lehrach H Reeves RH Yaspo ML Transcript level alterations reflect gene dosage effects across multiple tissues in a mouse model of down syndrome Genome Res 2004 14 7 1258 1267 10.1101/gr.1951304 15231742 PMC442140

[B10] Aït Yahya-Graison E Aubert J Dauphinot L Rivals I Prieur M Golfier G Rossier J Personnaz L Creau N Bléhaut H Robin S Delabar JM Potier MC Classification of human chromosome 21 gene-expression variations in Down syndrome: impact on disease phenotypes Am J Hum Genet 2007 81 3 475 91 10.1086/520000 17701894 PMC1950826

[B11] Chou CY Liu LY Chen CY Tsai CH Hwa HL Chang LY Lin YS Hsieh FJ Gene expression variation increase in trisomy 21 tissues Mamm Genome 2008 19 6 398 405 10.1007/s00335-008-9121-1 18594911

[B12] Gardiner K Davisson MT Crnic LS Building protein interaction maps for Down's syndrome Brief Funct Genomic Proteomic 2004 3 2 142 156 10.1093/bfgp/3.2.142 15355596

[B13] Shin JH Gulesserian T Weitzdoerfer R Fountoulakis M Lubec G Derangement of hypothetical proteins in fetal Down's syndrome brain Neurochem Res 2004 29 6 1307 1316 15176487 10.1023/b:nere.0000023617.49590.19

[B14] Seregaza Z Roubertoux PL Jamon M Soumireu-Mourat B Mouse models of cognitive disorders in trisomy 21: a review Behav Genet 2006 36 3 387 404 10.1007/s10519-006-9056-9 16523244

[B15] Gropp A Kolbus U Giers D Systematic approach to the study of trisomy in the mouse. II Cytogenet Cell Genet 1975 14 1 42 62 10.1159/000130318 1132247

[B16] Reeves RH Irving NG Moran TH Wohn A Kitt C Sisodia SS Schmidt C Bronson RT Davisson MT A mouse model for Down syndrome exhibits learning and behaviour deficits Nat Genet 1995 11 2 177 184 10.1038/ng1095-177 7550346

[B17] Sago H Carlson EJ Smith DJ Kilbridge J Rubin EM Mobley WC Epstein CJ Huang TT Ts1Cje, a partial trisomy 16 mouse model for Down syndrome, exhibits learning and behavioral abnormalities Proc Natl Acad Sci USA 1998 95 11 6256 6261 10.1073/pnas.95.11.6256 9600952 PMC27649

[B18] Gahtan E Auerbach JM Groner Y Segal M Reversible impairment of long-term potentiation in transgenic Cu/Zn-SOD mice Eur J Neurosci 1998 10 2 538 544 10.1046/j.1460-9568.1998.00058.x 9749716

[B19] FitzPatrick DR Ramsay J McGill NI Shade M Carothers AD Hastie ND Transcriptome analysis of human autosomal trisomy Hum Mol Genet 2002 11 26 3249 3256 10.1093/hmg/11.26.3249 12471051

[B20] Mao R Zielke CL Zielke HR Pevsner J Global up-regulation of chromosome 21 gene expression in the developing Down syndrome brain Genomics 2003 81 5 457 467 10.1016/S0888-7543(03)00035-1 12706104

[B21] Chrast R Scott HS Papasavvas MP Rossier C Antonarakis ES Barras C Davisson MT Schmidt C Estivill X Dierssen M Pritchard M Antonarakis SE The mouse brain transcriptome by SAGE: differences in gene expression between P30 brains of the partial trisomy 16 mouse model of Down syndrome (Ts65Dn) and normals Genome Res 2000 10 12 2006 2021 10.1101/gr.10.12.2006 11116095 PMC313062

[B22] Saran NG Pletcher MT Natale JE Cheng Y Reeves RH Global disruption of the cerebellar transcriptome in a Down syndrome mouse model Hum Mol Genet 2003 12 16 2013 2019 10.1093/hmg/ddg217 12913072

[B23] Amano K Sago H Uchikawa C Suzuki T Kotliarova SE Nukina N Epstein CJ Yamakawa K Dosage-dependent over-expression of genes in the trisomic region of Ts1Cje mouse model for Down syndrome Hum Mol Genet 2004 13 13 1333 1340 10.1093/hmg/ddh154 15138197

[B24] Rhodes DR Chinnaiyan AM Integrative analysis of the cancer transcriptome Nat Genet 2005 37 Suppl S31 37 10.1038/ng1570 15920528

[B25] Bertram L McQueen MB Mullin K Blacker D Tanzi RE Systematic meta-analyses of Alzheimer disease genetic association studies: the AlzGene database Nat Genet 2007 39 1 17 23 10.1038/ng1934 17192785

[B26] Rasche A Al-Hasani H Herwig R Meta-analysis approach identifies candidate genes and associated molecular networks for type-2 diabetes mellitus BMC Genomics 2008 9 310 10.1186/1471-2164-9-310 18590522 PMC2515154

[B27] Sbai O Devi TS Melone MA Feron F Khrestchatisky M Singh LP Perrone L RAGE-TXNIP axis is required for S100B-promoted Schwann cell migration, fibronectin expression and cytokine secretion J Cell Sci 2010 123 Pt24 4332 9 21098642 10.1242/jcs.074674

[B28] Parikh H Carlsson E Chutkow WA Johansson LE Storgaard H Poulsen P Saxena R Ladd C Schulze PC Mazzini MJ Jensen CB Krook A Björnholm M Tornqvist H Zierath JR Ridderstråle M Altshuler D Lee RT Vaag A Groop LC Mootha VK TXNIP regulates peripheral glucose metabolism in humans PLoS Med 2007 4 5 e158. 17472435 10.1371/journal.pmed.0040158 PMC1858708

[B29] Yamawaki H Haendeler J Berk BC Thioredoxin: a key regulator of cardiovascular homeostasis Circ Res 2003 93 11 1029 1033 10.1161/01.RES.0000102869.39150.23 14645133

[B30] Austin C Does oxidative damage contribute to the generation of leukemia? Leuk Res 2009 33 10 1297 10.1016/j.leukres.2009.04.038 19560202

[B31] Flicek P Aken BL Ballester B Beal K Bragin E Brent S Chen Y Clapham P Coates G Fairley S Fitzgerald S Fernandez-Banet J Gordon L Gräf S Haider S Hammond M Howe K Jenkinson A Johnson N Kähäri A Keefe D Keenan S Kinsella R Kokocinski F Koscielny G Kulesha E Lawson D Longden I Massingham T McLaren W Ensembl's 10th year Nucleic Acids Res 38 Database D557 562 10.1093/nar/gkp972 PMC2808936 19906699

[B32] Dierssen M de Lagran MM DYRK1A (dual-specificity tyrosine-phosphorylated and -regulated kinase 1A): a gene with dosage effect during development and neurogenesis ScientificWorldJournal 2006 6 1911 1922 17205196 10.1100/tsw.2006.319 PMC5917402

[B33] Edwards H Xie C LaFiura KM Dombkowski AA Buck SA Boerner JL Taub JW Matherly LH Ge Y RUNX1 regulates phosphoinositide 3-kinase/AKT pathway: role in chemotherapy sensitivity in acute megakaryocytic leukemia Blood 2009 114 13 2744 2752 19638627 10.1182/blood-2008-09-179812 PMC2756129

[B34] Gardiner K Transcriptional dysregulation in Down syndrome: predictions for altered protein complex stoichiometries and post-translational modifications, and consequences for learning/behavior genes ELK, CREB, and the estrogen and glucocorticoid receptors Behav Genet 2006 36 3 439 453 10.1007/s10519-006-9051-1 16502135

[B35] Butler C Knox AJ Bowersox J Forbes S Patterson D The production of transgenic mice expressing human cystathionine beta-synthase to study Down syndrome Behav Genet 2006 36 3 429 38 10.1007/s10519-006-9046-y 16541333

[B36] Belichenko PV Kleschevnikov AM Salehi A Epstein CJ Mobley WC Synaptic and cognitive abnormalities in mouse models of Down syndrome: exploring genotype-phenotype relationships J Comp Neurol 2007 504 4 329 345 10.1002/cne.21433 17663443

[B37] Ronan A Fagan K Christie L Conroy J Nowak NJ Turner G Familial 4.3 Mb duplication of 21q22 sheds new light on the Down syndrome critical region J Med Genet 2007 44 7 448 451 10.1136/jmg.2006.047373 17237124 PMC2598003

[B38] Korbel JO Tirosh-Wagner T Urban AE Chen XN Kasowski M Dai L Grubert F Erdman C Gao MC Lange K Sobel EM Barlow GM Aylsworth AS Carpenter NJ Clark RD Cohen MY Doran E Falik-Zaccai T Lewin SO Lott IT McGillivray BC Moeschler JB Pettenati MJ Pueschel SM Rao KW Shaffer LG Shohat M Van Riper AJ Warburton D Weissman S The genetic architecture of Down syndrome phenotypes revealed by high-resolution analysis of human segmental trisomies Proc Natl Acad Sci USA 2009 106 29 12031 12036 10.1073/pnas.0813248106 19597142 PMC2709665

[B39] Lyle R Béna F Gagos S Gehrig C Lopez G Schinzel A Lespinasse J Bottani A Dahoun S Taine L Doco-Fenzy M Cornillet-Lefèbvre P Pelet A Lyonnet S Toutain A Colleaux L Horst J Kennerknecht I Wakamatsu N Descartes M Franklin JC Florentin-Arar L Kitsiou S Aït Yahya-Graison E Costantine M Sinet PM Delabar JM Antonarakis SE Genotype-phenotype correlations in Down syndrome identified by array CGH in 30 cases of partial trisomy and partial monosomy chromosome 21 Eur J Hum Genet 2009 17 4 454 66 10.1038/ejhg.2008.214 19002211 PMC2986205

[B40] Kamburov A Wierling C Lehrach H Herwig R ConsensusPathDB--a database for integrating human functional interaction networks Nucleic Acids Res 2009 37 Database D623 628 10.1093/nar/gkn698 PMC2686562 18940869

[B41] Subramanian A Kuehn H Gould J Tamayo P Mesirov JP GSEA-P: a desktop application for Gene Set Enrichment Analysis Bioinformatics 2007 23 23 3251 3253 10.1093/bioinformatics/btm369 17644558

[B42] Wingender E Kel AE Kel OV Karas H Heinemeyer T Dietze P Knuppel R Romaschenko AG Kolchanov NA TRANSFAC, TRRD and COMPEL: towards a federated database system on transcriptional regulation Nucleic Acids Res 1997 25 1 265 268 10.1093/nar/25.1.265 9016550 PMC146363

[B43] Linhart C Halperin Y Shamir R Transcription factor and microRNA motif discovery: the Amadeus platform and a compendium of metazoan target sets Genome Res 2008 18 7 1180 1189 10.1101/gr.076117.108 18411406 PMC2493407

[B44] Choi KH Elashoff M Higgs BW Song J Kim S Sabunciyan S Diglisic S Yolken RH Knable MB Torrey EF Webster MJ Putative psychosis genes in the prefrontal cortex: combined analysis of gene expression microarrays BMC Psychiatry 2008 8 87 10.1186/1471-244X-8-87 18992145 PMC2585075

[B45] Choi JW Herr DR Noguchi K Yung YC Lee CW Mutoh T Lin ME Teo ST Park KE Mosley AN Chun J LPA receptors: subtypes and biological actions Annu Rev Pharmacol Toxicol 50 157 186 10.1146/annurev.pharmtox.010909.105753 20055701

[B46] Kakinuma N Zhu Y Wang Y Roy BC Kiyama R Kank proteins: structure, functions and diseases Cell Mol Life Sci 2009 66 16 2651 2659 10.1007/s00018-009-0038-y 19554261 PMC11115667

[B47] Allikmets R Dean M Bringing age-related macular degeneration into focus Nat Genet 2008 40 7 820 821 10.1038/ng0708-820 18583975

[B48] Esbensen AJ Health conditions associated with aging and end of life of adults with Down syndrome Int Rev Res Ment Retard 2010 39 C 107 126 21197120 10.1016/S0074-7750(10)39004-5 PMC3010180

[B49] Vis JC Duffels MG Winter MM Weijerman ME Cobben JM Huisman SA Mulder BJ Down syndrome: a cardiovascular perspective J Intellect Disabil Res 2009 53 5 419 25 10.1111/j.1365-2788.2009.01158.x 19228275

[B50] Damgaard T Knudsen LM Dahl IM Gimsing P Lodahl M Rasmussen T Regulation of the CD56 promoter and its association with proliferation, anti-apoptosis and clinical factors in multiple myeloma Leuk Lymphoma 2009 50 2 236 246 10.1080/10428190802699332 19235015

[B51] Wang CC Tsai MF Dai TH Hong TM Chan WK Chen JJ Yang PC Synergistic activation of the tumor suppressor, HLJ1, by the transcription factors YY1 and activator protein 1 Cancer Res 2007 67 10 4816 4826 10.1158/0008-5472.CAN-07-0504 17510411

[B52] Li W Wang C Juhn SK Ondrey FG Lin J Expression of fibroblast growth factor binding protein in head and neck cancer Arch Otolaryngol Head Neck Surg 2009 135 9 896 901 10.1001/archoto.2009.121 19770422 PMC2845968

[B53] Hayes JD Flanagan JU Jowsey IR Glutathione transferases Annu Rev Pharmacol Toxicol 2005 45 51 88 10.1146/annurev.pharmtox.45.120403.095857 15822171

[B54] Xavier AC Ge Y Taub JW Down syndrome and malignancies: a unique clinical relationship: a paper from the 2008 william beaumont hospital symposium on molecular pathology J Mol Diagn 2009 11 5 371 80 10.2353/jmoldx.2009.080132 19710397 PMC2729834

[B55] Reymond A Marigo V Yaylaoglu MB Leoni A Ucla C Scamuffa N Caccioppoli C Dermitzakis ET Lyle R Banfi S Eichele G Antonarakis SE Ballabio A Human chromosome 21 gene expression atlas in the mouse Nature 2002 5;420 6915 582 6 12466854 10.1038/nature01178

[B56] Moldrich RX Dauphinot L Laffaire J Rossier J Potier MC Down syndrome gene dosage imbalance on cerebellum development Prog Neurobiol 2007 82 2 87 94 10.1016/j.pneurobio.2007.02.006 17408845

[B57] Dauphinot L Lyle R Rivals I Dang MT Moldrich RX Golfier G Ettwiller L Toyama K Rossier J Personnaz L Antonarakis SE Epstein CJ Sinet PM Potier MC The cerebellar transcriptome during postnatal development of the Ts1Cje mouse, a segmental trisomy model for Down syndrome Hum Mol Genet 2005 14 3 373 384 15590701 10.1093/hmg/ddi033

[B58] Sommer CA Pavarino-Bertelli EC Goloni-Bertollo EM Henrique-Silva F Identification of dysregulated genes in lymphocytes from children with Down syndrome Genome 2008 51 1 19 29 10.1139/G07-100 18356936

[B59] Barrett T Troup DB Wilhite SE Ledoux P Rudnev D Evangelista C Kim IF Soboleva A Tomashevsky M Marshall KA Phillippy KH Sherman PM Muertter RN Holko M Ayanbule O Yefanov A Soboleva A NCBI GEO: archive for high-throughput functional genomic data Nucleic Acids Res 2009 37 Database D885 890 10.1093/nar/gkn764 PMC2686538 18940857

[B60] Parkinson H Kapushesky M Kolesnikov N Rustici G Shojatalab M Abeygunawardena N Berube H Dylag M Emam I Farne A Holloway E Lukk M Malone J Mani R Pilicheva E Rayner TF Rezwan F Sharma A Williams E Bradley XZ Adamusiak T Brandizi M Burdett T Coulson R Krestyaninova M Kurnosov P Maguire E Neogi SG Rocca-Serra P Sansone SA ArrayExpress update--from an archive of functional genomics experiments to the atlas of gene expression Nucleic Acids Res 2009 37 Database D868 872 10.1093/nar/gkn889 PMC2686529 19015125

