# Supplementary figures and images for: Meta-analysis of heterogeneous Down Syndrome data reveals consistent genome-wide dosage effects related to neurological processes
Source: BMC Genomics. 2011 May 11;12:229. doi: 10.1186/1471-2164-12-229 (PMC3110572; doi:10.1186/1471-2164-12-229)

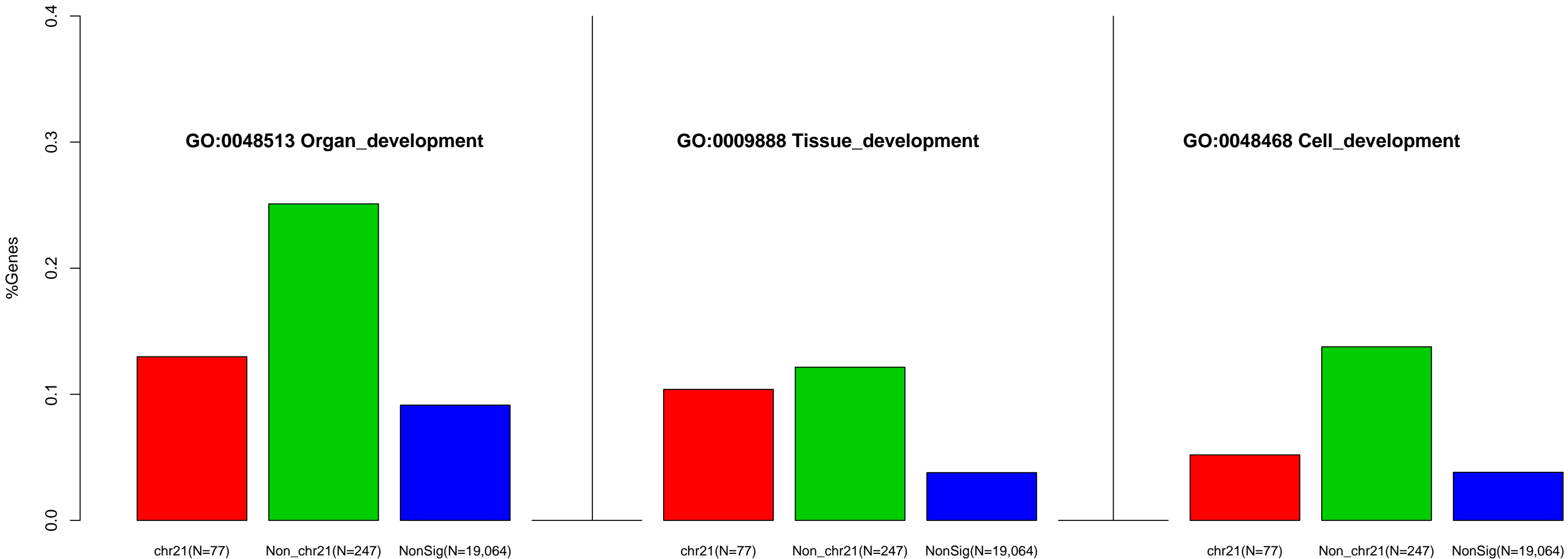

Supplement: Additional file 2 — Figure S1. Enrichment of GO categories for organ, tissue and cell development with respect to the significant HSA21 genes (red bars), the significant non-HSA21 genes (green bars) and the non-significant genes (blue bars). [file 1471-2164-12-229-S2.PDF]

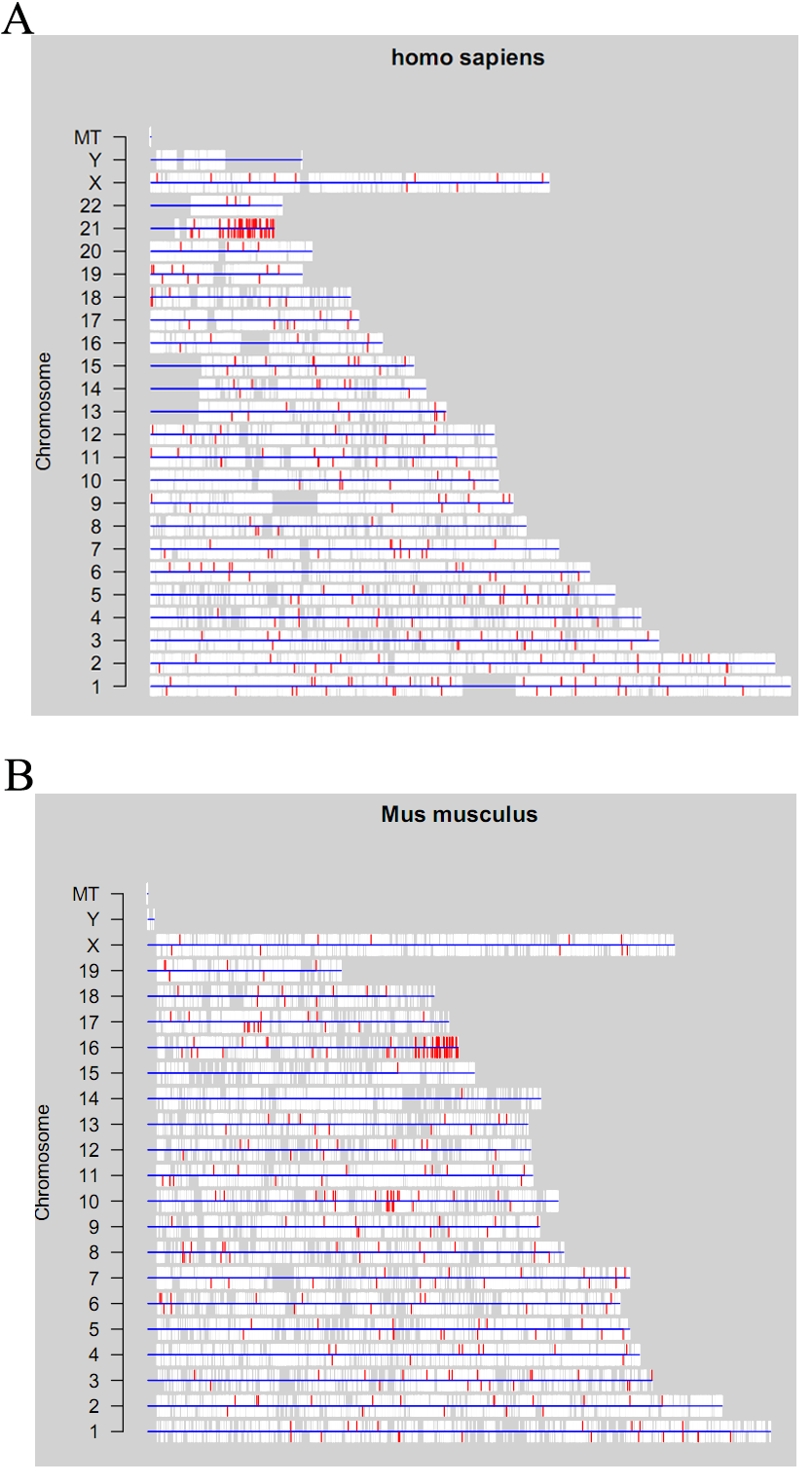

Supplement: Additional file 3 — Figure S2. Genomic location of DS dosage effects in A) human B) mouse. Significant genes are marked in red, non-significant genes in white. [file 1471-2164-12-229-S3.TIFF]

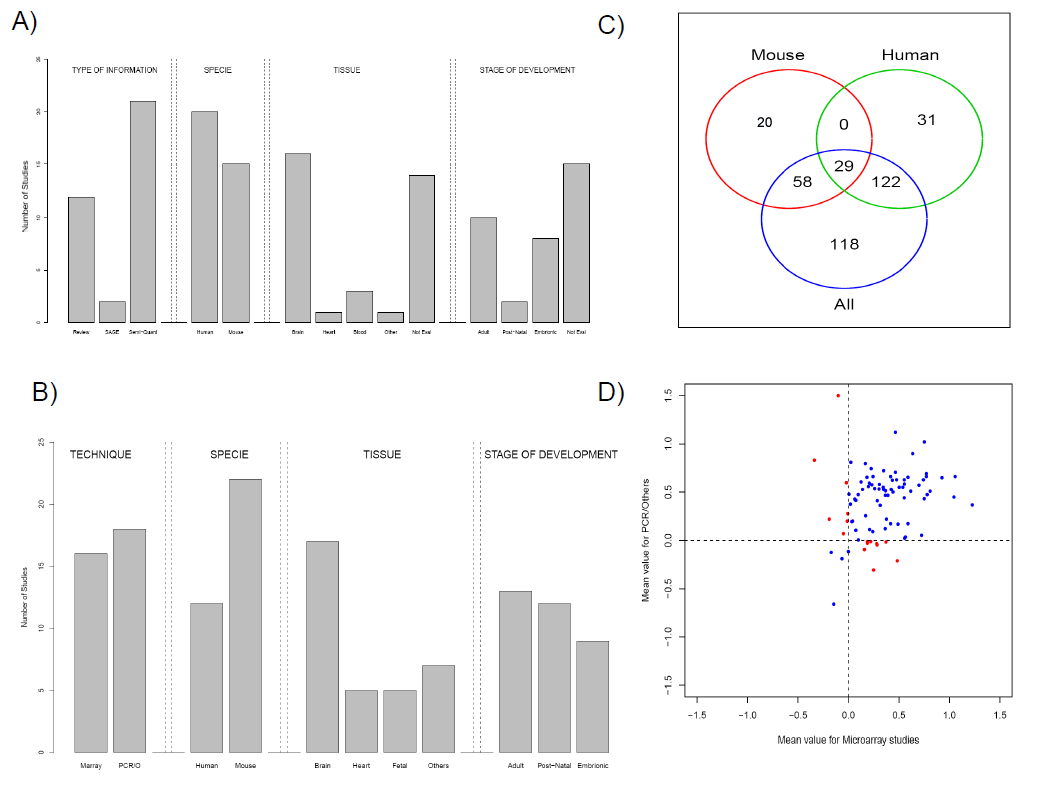

Supplement: Additional file 4 — Figure S3. A) Categorization of the 35 qualitative studies, B) Categorization of the 34 quantitative studies. C) Venn diagram of dosage effects detected with mouse and human data alone and with the combination of all data, D) correlation between average PCR and microarray values for the detected 324 dosage effects. [file 1471-2164-12-229-S4.TIFF]

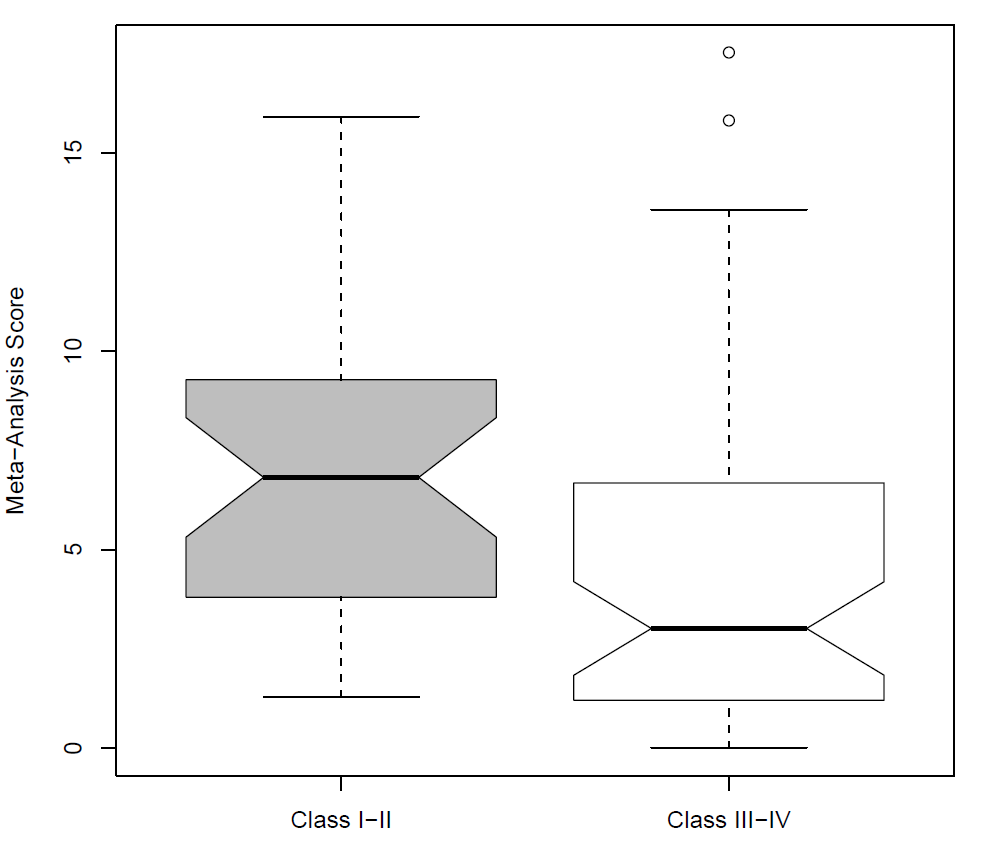

Supplement: Additional file 5 — Figure S4. Cross-validation with DS dosage effects detected with an HSA21 microarray [54]. Box-plots of meta-analysis scores (Y-axis) for class I and II (dosage effects) and class III and IV (compensation and variable expression) genes as judged by the authors. [file 1471-2164-12-229-S5.TIFF]
